# How Does Environmental Information Disclosure Affect Public Health? Evidence from the New Ambient Air Quality Standards

**DOI:** 10.3390/ijerph192215141

**Published:** 2022-11-17

**Authors:** Xiang Zhang, Yanan Wang, Zongyi Zhang, Hongyu Long

**Affiliations:** 1 Accounting School, Chongqing University of Technology, Chongqing 400054, China; 2 School of Economics and Business Administration, Chongqing University, Chongqing 400030, China; 3 Nanyang Technopreneurship Centre, Nanyang Technological University, Singapore 639798, Singapore

**Keywords:** Ambient Air Quality Standards, environmental information disclosure, public health, mental health, physical health

## Abstract

Using a quasi-natural experiment of the implementation of the new Ambient Air Quality Standards in China, this paper assessed the impact of environmental information disclosure on public health. Our empirical results showed that environmental information disclosure (EID) largely improved both physical health and mental health. Moreover, we further investigated the air pollution channel, and the empirical results showed that EID could reduce the concentration of PM_2.5_, which could cause an increase in public health as the concentration of PM_2.5_ decreases. In addition, in terms of individual characteristics, the impact of EID was larger for men, people living in the countryside and people older than 60. In terms of the heterogeneity of cities, the impact of EID was larger in cities with higher public environmental concerns, and the impact of EID was more pronounced in core cities. For regional heterogeneity, the impact of EID on physical health was more pronounced in more developed regions, whereas the impact EID on mental health was higher in less developed regions.

## 1. Introduction

With continuous substantial growth over the last forty years, China has become the second largest economy in the world [1]. Economic growth in China is mainly driven by high capital input and low labor costs, and this growth path features low resource allocation efficiency [2], which causes the great consumption of resources and energy. Thus, the economic growth in China also leads to numerous environmental issues, especially air pollution [3]. Numerous studies have proven the adverse effects of air pollution on human health [4,5,6], which further impacts the sustainable growth of China.

To improve China’s environmental quality, since the 1980s, its government has implemented a series of regulation policies. However, environmental regulation in the early stages mainly focused on sulfur dioxide (SO_2_) emissions, whose effect is very limited [7]. Meanwhile, for the past few decades, high economic growth has not only increased pollutant emissions, but the composition of the air pollution has also changed; more specifically, high concentrations of PM_2.5_ (PM_2.5_ is fine particulate matter, which is especially harmful and can easily penetrate a person’s lung, bloodstream and even brain) have been found in the Beijing–Tianjin–Hebei region and the Yangtze River Delta region. As a result, to address this issue, the new Ambient Air Quality Standards (AAQS) have been implemented by The Ministry of Environmental Protection of China (MEPC) since 2012.

Unlike other environmental regulation policies with punishments or political interventions, AAQS concerns the environmental information disclosure (EID) of the concentrations of pollutants in ambient air. A total of 190 cities’ hourly monitoring data of air pollution indexes are publicized in the national web platform with AAQS [8], and this provides an important basis for environmental protection authorities and relevant departments to manage ambient air quality and formulate pollutant emission standards. By comparing the air pollution indexes before and after the implementation of AAQS, using the method of descriptive statistics, numerous studies have investigated the impact of AAQS on air pollution. The annual average concentrations of total suspended particulates, PM_2.5_ and SO_2_, have shown decreasing trends since the implementation of the AAQS [9,10]. Using the difference-in-differences method, evidence shows that the concentration of PM_2.5_ and emissions of SO_2_ in pilot cities have also since been significantly reduced [11].

However, the causal inference of the impact of environmental information disclosure on public health has never been investigated. To fill the gap, this paper attempted to explore the relationship between environmental information disclosure and public health with a quasi-natural experiment in China, as well as to explore the mediation effect of air quality. Moreover, we also investigated the moderating effect of personal characteristics and public environmental concerns. Finally, heterogeneity in terms of cities and regions was examined as well.

This study contributes to current research in the following aspects. First, taking AAQS as a quasi-natural experiment, this paper investigated the impact of EID on public health, whereas current studies have only focused on the relationship between AAQS and air pollution. Moreover, most of the current studies have generally compared the changes in ambient air quality before and after the implementation of AAQS using a descriptive statistics analysis, and the causal inference technique has not been applied; thus, the reliability of the conclusions is limited. The difference-in-differences method was employed in this paper, and the findings are consistent with numerous robustness checks. Second, two categories of public health were studied: physical health and mental health. Using the survey data from the China Health and Retirement Longitudinal Study (CHARLS), the two indices of acute shock and chronic shock were constructed to measure physical health, and the three indices of situational memory, mental cognition and depression self-assessment were constructed to measure mental health. Third, we further investigated the impact mechanism of EID on public health. Specifically, the air pollution channel was studied. Our results show that, proxied by PM_2.5_, air pollution decreased with EID, and subsequently the reduction in air pollution improved public health. Finally, the analysis of heterogeneity was conducted from the perspective of individual characteristics, namely, the heterogeneity of cities and regional heterogeneity. Specifically, for individual characteristics, gender, residence and age were considered. In terms of the heterogeneity of cities, public environmental concerns and the political level of cities were studied. For regional heterogeneity, economic development was investigated.

## 2. Policy Background and Research Hypothesis

### 2.1. Policy Background

**Figure 1:**
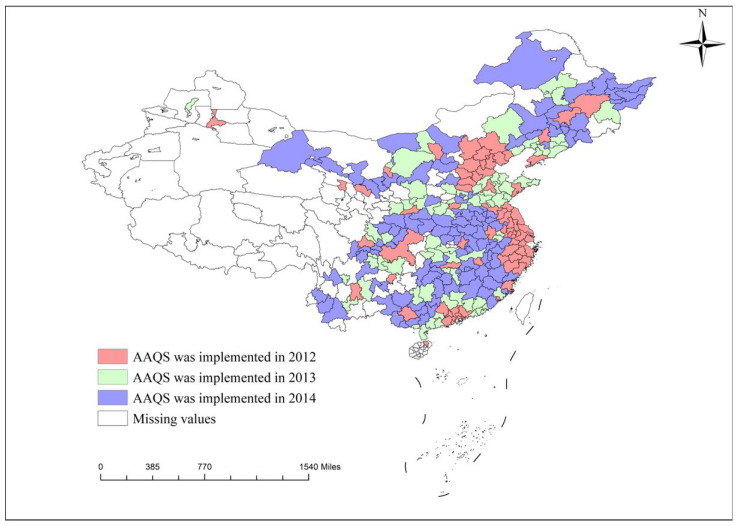
Spatial distribution of AAQS implementation cities.

Similar to other countries, the environmental policies adopted by the Chinese government began with legal constraints based on administrative orders [12]. Early legal regulations often had a significant environmental improvement effect. However, as productivity developed and production demand increased, many companies began to bypass environmental regulations and commit production violations, which led to a sharp decline in environmental quality [13,14]. To continuously improve the environment, promote government regulation, and strengthen public oversight, China has begun to focus on the role of environmental information disclosure in pollution control and to meet public demand for environmental information disclosure [15]. The Ambient Air Quality Standard was promulgated in 2012 as a policy product of this background. This standard requires that air quality data must be released to the public in a uniform, real-time, and comprehensive manner [10]. In addition, the government will announce in four batches the monitoring stations set up in the corresponding cities nationwide for the establishment of the ambient air quality network. In May 2012, the government announced the first phase of 496 monitoring stations. The first batch of cities included 74 prefecture-level cities in core regions such as Beijing, Tianjin, Hebei, Yangtze River Delta and Pearl River Delta, as well as municipalities directly under the control of the central government and provincial capitals. The second batch of 388 monitoring stations was announced in March 2013, which included 87 prefecture-level cities such as key national environmental protection cities and model cities. The third batch, consisting of 117 other prefecture-level cities, was announced in May 2014 and contained 552 monitoring sites; the detailed names of cities are shown in Table 1, and the spatial distribution of AAQS implementation cities is presented in Figure 1. In January 2015, the government announced the full rollout of the Ambient Air Quality Standards, which contain a total of 1436 monitoring sites in 338 cities at the prefecture level and above, nationwide. The announcement of the Ambient Air Quality Standards has facilitated public participation in air quality monitoring, while enriching the government’s means of combating air pollution and strengthening the environmental awareness of enterprises [10]. As the disclosure of environmental information raises society’s concern about air quality, this may change the air pollution situation, especially regarding the PM_2.5_ concentration.

### 2.2. Research Hypothesis

Different pollutants can cause different diseases. On one hand, air pollutants, such as SO_2_, PM_10_, PM_2.5_, etc., lead to an increase in the prevalence of respiratory diseases and may even cause an increase in death and reduce expected lifetimes. On the other hand, in some diseases related to air pollution, such as cardiovascular diseases and respiratory diseases, the same air pollutant may cause different mortality rates [16].

To further monitor air quality, the new AAQS was implemented by The MEPC in 2012. The new AAQS emphasizes the control of the concentration of PM_2.5_; air quality data must be released to the public in real time [10], thus, the AAQS improves environmental information disclosure on the concentrations of pollutants in ambient air [8]. Current studies show that air pollution has decreased with the implementation of the AAQS, and with the method of descriptive statistics, the concentrations of PM_2.5_ and SO_2_ have also since shown a decreasing trend [9,10]. With the causal inference method, it is proven that this implementation significantly reduces the air pollution proxied by PM_2.5_ concentration and SO_2_ emission [11].

Moreover, based on the China Family Panel Studies (CFPS) database, evidence shows that the reduction in air pollution significantly increases self-reported health and reduces the infant mortality rate [17]. In addition, the reduction in ambient air pollutant exposures would contribute to a decrease in dementia deaths by 4.17%, according to the WHO air quality guidelines, and by 0.39% according to the AAQS in China [18]. Chronic exposure to air pollution is proven to be correlated with cardiovascular [19], pulmonary [20], and metabolic dysfunction [21], resulting in an increase in premature mortality.

Consequently, once the disclosed air quality becomes too poor, the public can intervene by complaining directly to the government; in response, the government will force firms to reduce the emission of pollutants to improve the air quality. Moreover, as a result, the public will also pay more attention to their outdoor activities, thus, reducing their exposure to air pollution, which is harmful to their health. As a result, the improved air quality helps improve public health.

In terms of mental health, air pollution crosses the blood–brain barrier, which leads to increased neuroinflammation and neurotoxicity, and this results in pathological changes [22]. Recently, air pollution has been found to be associated with depression in older populations [23], psychiatric emergency room visits [24], and mental health disorders [25]. Moreover, the exposure of pregnant women to air pollution increases the probability of mental disorders complicating pregnancy, and the same results are found for depression [26]. Thus, mental health is also highly correlated with air quality. More importantly, health issues mainly originate from the high pressures of work in China, while this problem is rare in other countries. This kind of pressure may cause serious mental health issues, which environmental pollution will enhance [27]. Therefore, the improvement of air quality will significantly relieve people’s work pressure, and thus, mental health will be improved.

As a result, we propose the following hypotheses:

**H1**:  *Public health increases with environmental information disclosure.*

**H2**:  *Air pollution decreases with environmental information disclosure, and the reduction in air pollution will improve public health.*

## 3. Methods and Data

### 3.1. Data

This paper uses data for the years of 2011, 2013, 2015, and 2018 from the three-wave China Health and Retirement Longitudinal Survey (CHARLS); the data cover adults aged 45 years or older. With the Probabilities Proportional to Size (PPS) method, the CHARLS randomly selects 150 cities from 30 provinces. Based on the population, the CHARLS survey data contain 450 communities. CHARLS provides a large amount of information on public health related to the rapid ageing of the population in China [28]. Data on region-wide variables are obtained from the Chinese City Statistics Database (CCSD) of the Chinese Research Data Services (CNRDS) (Data source: https://www.cnrds.com/Home/Index#/FinanceDatabase/DB/CCSD, accessed on 1 October 2022) Platform and the China Urban Statistical Yearbook.

### 3.2. Econometric Model

#### 3.2.1. Baseline Model

To assess the impact of environmental information disclosure on public health, this paper constructs the following baseline DID model:

(1) $$y_{i,r,t}=\beta_{0}+\beta_{1}\times EID_{r,t}+Control_{i,r,t}+\lambda_{t}+\gamma_{r}+\varepsilon_{i,r,t}$$

(2) $$EID_{r,t}=\left\{\begin{array}{l} 0,\;EID\;is\;not\;implemented.\\ 1,EID\;is\;implemented\;in\;city\;r\;at\;year\;t.\; \end{array}\right.$$

where *y*_*i*__,__*r*__,__*t*_ denotes the public health of individuals *i* in city *r* at year *t*; specifically, physical health and mental health are used to measure public health. *EID_r_*_,*t*_ denotes the implementation of the new Ambient Air Quality Standards in 2012, and the estimated coefficient *β*_1_ denotes the average treatment effect of EID on public health. *Control_i_*_,*r*,*t*_ denotes a series of control variables, *λ_t_* denotes time fixed effect, *γ_r_* denotes city fixed effect, and *ε_i_*_,*r*,*n*,*t*_ denotes error term.

#### 3.2.2. Mediating Effect Model

To further test hypothesis 2, i.e., the mediating effect of air pollution, the following mediating effect model is constructed:

(3) $$y_{i,r,t}=\alpha_{0}+\alpha_{1}\times EID_{r,t}+Control_{i,r,t}+\lambda_{t}+\gamma_{r}+\varepsilon_{i,r,t}$$

(4) $$PM_{2.5_{r,t}}=\beta_{0}+\beta_{1}\times EID_{r,t}+\lambda_{t}+\gamma_{r}+\varepsilon_{r,t}$$

(5) $$y_{i,r,t}=\delta_{0}+\delta_{1}\times EID_{r,t}+\delta_{2}\times PM_{2.5_{r,t}}+Control_{i,r,t}+\lambda_{t}+\gamma_{r}+\varepsilon_{i,r,t}$$

where *PM*_2.5*r*,*t*_ denotes the PM_2.5_ concentration of city r in year t. If *β*_1_ in Equation (4) passes the 10% significance level test, it indicates that environmental information disclosure has a significant impact on PM_2.5_ concentration. On this basis, if both *β*_1_ and *β*_2_ in Equation (5) pass the 10% significance level test, it indicates that the impact of environmental information disclosure on public health is mediated by PM_2.5_ concentration.

#### 3.2.3. Moderating Effect Model

In this section, we attempt to investigate the moderating effect. The following moderating effect model is constructed:

(6) $$y_{i,r,t}=\alpha_{0}+\alpha_{1}\times X_{r,t}+Control_{i,r,t}+\lambda_{t}+\gamma_{r}+\varepsilon_{i,r,t}$$

(7) $$y_{i,r,t}=\beta_{0}+\beta_{1}\times EID_{r,t}+\beta_{2}\times X_{r,t}+\beta_{3}\times X_{r,t}\times EID_{r,t}+Control_{i,r,t}+\lambda_{t}+\gamma_{r}+\varepsilon_{i,r,t}$$

where *X_i_*_,*r*,*t*_ denotes the moderating variables. Three categories of moderating variables are employed, including individual characteristics, city-level variables, and regional variables. Specifically, gender, residence, and age are employed for individual characteristics. Public environmental concerns and the political level of cities are studied to analyze the heterogeneity of cities, while economic development is used for regional heterogeneity. Detailed definitions of the moderating variables are presented in Table 2.

### 3.3. Measurement of Variables

#### 3.3.1. Public Health

The dependent variable in this paper is public health; following [29], two dimensions of public health are measured: physical health and mental health. To measure physical health, two indices are used: acute shock and chronic shock. More specifically, acute shock is constructed as follows: interviewees are asked whether they suffer from cardiopathy, apoplexy, or cancer. Each disease gives 1 point; if the interviewee is suffering from this disease, then they are scored 1 point, 0 if not. Thus, the score of acute shock is banded between 0 and 3, and the larger the acute shock, the worse the interviewee’s state of physical health. Likewise, chronic shock is constructed the same way, and 9 chronic diseases are enquired, including hypertension, dyslipidemia, diabetes, chronic lung diseases, liver diseases, kidney diseases, gastric diseases, arthritis, and asthma. Thus, the score of chronic shock is banded between 0 and 9, and the larger the chronic shock, the worse the interviewee’s state of physical health. Then, each index is standardized and transformed until it is banded between 0 and 1; finally, they are summed up with a weighted average to form the physical health index. Thus, physical health is computed as follows:

Physical health = (acute shock/3 + chronic shock/9)/2

For mental health, 3 sub-indexes are constructed: situational memory, mental cognition, and depression self-assessment. Situational memory is constructed as follows: First, the interviewee is given 10 words, which they must repeat immediately; each word gives 1 point if the interviewee repeats correctly; otherwise, they score 0. After a few minutes, the interviewee is asked to repeat the 10 words again; likewise, each word gives 1 point if it is correctly repeated and 0 otherwise. Thus, the maximum score of situational memory is 20, and the minimum is 0 if the interviewee cannot remember any words. The larger the situational memory, the better the interviewee’s state of mental health. In terms of mental cognition, interviewees are asked to answer a total of 12 questions, regarding subtraction arithmetic, current season and time, and graph identification. Each question scores 1 point for a correct answer. Higher values of this indicator indicate higher levels of mental cognition. In terms of depression self-assessment, 10 questions are asked to interviewees about their feelings and behavior in the last week; 4 options are given for each question, denoting a score from 1 to 4. Each question is scored from 1 to 4. A score of 1 indicates the best self-assessment and a score of 4 indicates the worst self-assessment. The sum of the score for 10 questions is banded between 10 and 40, and the larger the depression self-assessment, the worse the interviewee’s state of mental health. Likewise, each index of mental health is standardized and summed up with weighted averages to form the mental health index; thus, mental health is computed as follows:

Mental health = [Situational memory/20 + mental cognition/12 + (depression self-assessment − 10)/3]/3

#### 3.3.2. Independent Variables

The independent variable in this paper is the implementation of EID. According to Equation (2), the sample after the implementation of EID takes the value of 1, and the sample before the implementation of EID takes the value of 0.

Meanwhile, a series of variables are controlled in this paper, including age (lnage), gender (gender), marital status (marital), residence properties (residence), health cost (lncost), sanitary environment (toilet), and domestic water (water). The measurement and descriptive statistics of each variable are shown in Table 3.

## 4. Empirical Results

### 4.1. Results of Baseline Regressions

The results of the impact of EID on public health are presented in Table 4; columns 1 and 2 report the results for physical health, and the results for mental health are presented in columns 3 and 4. City and year fixed effects are controlled for all specifications.

In Table 4, the coefficients of EID in columns 1 and 2 are 0.039 and 0.041, respectively; they are both statistically significant at the 1% level, which indicates that EID significantly improves public physical health. The coefficients of EID in columns 3 and 4 are 0.015 and 0.009, respectively, and both are statistically significant at the 1% level, which indicates that EID significantly improves public mental health. Based on the above results, after the individual features and the city and year fixed effects are controlled, EID is shown to have a positive impact on public health—the positive effects on physical health and mental health are 4.1% and 0.9%, respectively.

We further assess the impact of EID on each sub-index of physical health and mental health. Empirical results are reported in Table 5.

Table 5 shows that, in terms of physical health, the coefficients of EID in columns 1 and 2 are −0.050 and −0.597, respectively; they are both statistically significant at the 1% level, which indicates that EID significantly reduces the prevalence rate of acute shock and chronic shock by 5% and 59.7%. For mental health, the coefficients of EID in columns 3 and 5 are 0.145 and −0.253, respectively; they are both statistically significant at the 1% level, and this indicates that EID significantly improves situational memory by 14.5% and significantly reduces depression self-assessment by 25.3%. However, the impact of EID on mental cognition is statistically insignificant at the 10% level. Based on the above results, we find that EID significantly reduces the prevalence rate of acute disease, chronic disease, and depressive disorders. Moreover, EID significantly improves the public’s capacity for situational memory.

### 4.2. Impact Mechanism between EID and Public Health

According to H2, the impact of EID on public health is mediated by air pollution, and we employ the concentration of PM_2.5_ as a proxy for air pollution. Empirical results are presented in Table 6. Column 1 presents the results of the impact of EID on PM_2.5_, and columns 2 and 3 show the results of the mediation effect for physical health and mental health, respectively.

In Table 6, the Z-score of the Sobel test is −10.88 for physical health, which indicates that the mediation effect of air pollution is significant for physical health. Likewise, the Z-score of the Sobel test is 22.89 for mental health; thus, the mediation effect of air pollution is also significant for mental health. The results in column 1 show that the coefficient of EID is −0.201, which is significant at the 1% level. This indicates that EID significantly reduces the concentration of PM_2.5_. The coefficients of PM_2.5_ in columns 2 and 3 are −0.045 and −0.080, respectively, which are both significant at the 1% level. The results show that physical health and mental health are improved as PM_2.5_ decreases. Based on the findings, we claim that the impact of EID on public health is mediated by air pollution.

### 4.3. Heterogeneity Analysis

#### 4.3.1. Individual Characteristics

According to the study in [29], the level of physical health and mental health differs across different genders, residences, and ages. Thus, the heterogeneity of individual features should be considered. The empirical results are presented in Table 7, Table 8 and Table 9. In terms of gender, we divided the sample into two subsamples: male and female. For residence, we also constructed two subsamples: people living in the countryside and those living in cities. Finally, people aged between 45 and 60 were included in one subsample, and another subsample contained people older than 60. Table 7 presents the results for gender, Table 8 reports the findings of residence, and the results for age are presented in Table 9. A Seemingly Unrelated Regression test was applied to test the differences between the two groups.

According to the results of Table 7, the coefficients of EID are both significantly positive at the 1% level in columns 1 and 2, and the coefficient of EID for men is larger than for women, which indicates that the impact of EID on physical health for men is higher than for women, and the SUR test shows that the difference is significant. Likewise, the same results are found for mental health; the impact of EID on mental health for men is higher than for women, and the SUR test shows that the difference is significant.

Table 8 shows that the coefficients of EID are both significantly positive at the 1% level in columns 1 and 2. The coefficient of EID in column 1 is larger than the coefficient in column 2, which indicates that the impact of EID on physical health is higher for people living in the countryside than those living in cities, and the SUR test shows that the difference is significant. Moreover, the coefficient of EID in column 3 is insignificant, and it is significantly positive in column 4, which indicates that the impact of EID on mental health is higher for those living in cities than for people living in the countryside. The SUR test shows that the difference is significant. For these findings, the possible explanations are as follows. First, in some areas, especially in the north of China, heating in winter still mainly depends on coal, and this causes serious environmental issues, which further cause severe physical problems. People living in cities are usually covered by health insurance, and they usually pay more attention to their health. As a result, the impact of EID is higher for people living in the countryside than for those living in cities. Moreover, the health issues for people living in cities mainly originate from the high pressure of work in China, whereas this problem is rare in the countryside. This kind of pressure may cause serious mental health issues, and environmental pollution enhances these problems. Thus, the impact of EID on mental health is significantly higher for people living in cities.

According to the results in Table 9, the coefficients of EID are significantly positive at the 1% level in both columns 1 and 2, and the coefficient of EID is larger for people older than 60 than for those aged between 45 and 60, which indicates that the impact of EID on physical health for older people is significantly higher than for younger people, and the SUR test shows that the difference is significant. The same results are found for mental health, as the impact of EID on mental health is higher for people older than 60 than those aged between 45 and 60, and the SUR test shows that the difference is significant.

#### 4.3.2. Public Environmental Concerns

In this section, we investigate the moderating effect of public environmental concerns; the empirical results are presented in Table 10.

According to the results of Table 10, the interaction terms of EID and PEC are significantly positive at the 1% level in columns 1 and 2, which indicates that the impacts of EID on physical health and mental health for cities with higher public environmental concerns are 0.7% and 0.9% higher than for cities with lower public environmental concerns, respectively. The above results show that the impact of EID on public health is moderated by PEC, and this impact is more pronounced in cities with higher public environmental concerns.

#### 4.3.3. Group Analysis of Urban Administrative Rank and Economic Development Level

There exist large differences in terms of the development of cities in China, especially as regards urban administrative rank and economic development level. Generally, cities with higher urban administrative rank and larger economic development are endowed with more medical resources; thus, it is necessary to assess the heterogeneity of cities [30]. As regards urban administrative rank, provincial capitals, sub-provincial cities, and municipalities directly under the central government are determined as non-ordinary prefecture-level cities, and the others are defined as ordinary prefecture-level cities. The results for urban administrative rank are presented in Table 11.

According to Table 11, the coefficients of EID are 0.075 and 0.037 in columns 1 and 2, respectively, and both are significantly positive at the 1% level. Likewise, the coefficients of EID are 0.018 and 0.011 in columns 3 and 4, respectively, and both are significantly positive at the 1% level. Thus, the results show that the impacts of EID on physical health and mental health are higher in non-ordinary prefecture-level cities. As a result, the impact of EID is higher in cities with higher urban administrative rank.

In terms of economic development level, we split the sample into three regions: eastern region, central region, and western region. The empirical results are presented in Table 12.

According to Table 12, the impacts of EID on physical health for the eastern region, central region, and western region are 0.047, 0.039, and 0.039, respectively, and they are all statistically significant at the 1% level. Moreover, the impacts of EID on mental health for these three regions are 0.003, 0.008, and 0.015, respectively, and they are all statistically significant at the 1% level. Based on the above results, the impact of EID on physical health for the eastern region is the highest, and the impact of EID on mental health for the western region is the highest. As a result, the impact of EID on physical health is higher in more developed regions, and the impact of EID on mental health is higher in less developed regions.

### 4.4. Robustness Tests

#### 4.4.1. Placebo Test

**Figure 2:**
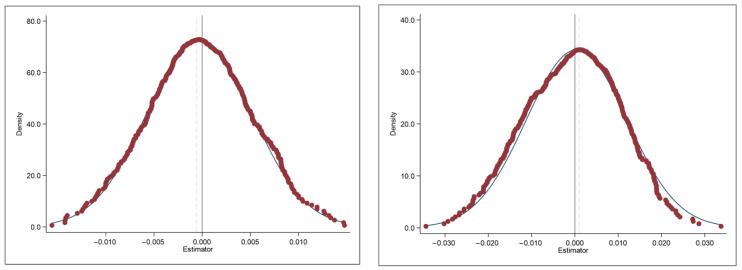
Results of placebo test. The treated group is randomly drawn 500 times in the control group by Monte Carlo simulation, and DID regression is performed. We plot the obtained regression coefficients as a distribution graph. This figure reports the results on public health (the dependent variable is physical health in the left panel and mental health in the right panel) of cities without environmental information disclosures as a dependent variable, presenting a normal distribution with an average value of 0.

The implementation of the AAQS may also impact the public health of cities that were previously without it; thus, the reliability may be affected. As a result, we employed Monte Carlo simulation as our placebo test, and the empirical results are presented in Figure 2. We randomly drew a sample from a control group to be the new treatment group, and then we re-estimated the model with the DID method. If the coefficients obtained after re-sampling are normally distributed with a mean of 0, then the results are robust. In this paper, we randomly drew 500 times for re-sampling, as we expected that the re-estimated coefficients would be normally distributed with zero mean. As a result, the improvement of public health originates from EID.

#### 4.4.2. Re-Estimation Based on PSM-DID

To ensure that the sample selection bias does not affect the reliability of the conclusions in this paper, we use the difference-in-difference model after propensity score matching (PSM-DID) for re-estimation. In this paper, we adopt the radius matching method with a radius of 0.04 to match the control group samples to the treatment group. The results of PSM-DID are shown in the following Table 13.

According to the results in Table 13, the coefficients of EID are 0.057 and 0.009, respectively, and both are statistically significant at the 1% level. Thus, the results of PSM-DID coincide with our findings of DID, which indicates that the EID significantly improves public health.

#### 4.4.3. Re-Estimation Using Different Dependent Variable

To ensure the robustness of our model, we employ an assessment of self-health to measure public health. The assessment of self-health involves the assessment of interviewees regarding their own health; the answers include excellent (five points), very good (four points), good (three points), fair (two points), and poor (one point). The estimation results are presented in Table 14; column 1 presents the results without any control variables, and control variables are added in column 2.

In Table 14, the coefficients of EID are 0.464 and 0.448 in columns 1 and 2, respectively, and both are statistically significant at the 1% level. The results are consistent; thus, our findings are robust.

#### 4.4.4. Control of Family-Related Economic Variables

Generally, an individual’s health situation is highly related to their economic conditions; thus, to isolate this impact, we add two control variables of family-related economic factors: natural logarithm of income (income) and natural logarithm of daily consumption (consumption). The empirical results are presented in Table 15 and Table 16. Table 15 shows the results for baseline regressions, and the results of mediation effect are reported in Table 16.

The coefficients of EID are significantly positive in columns 1 to 4 in Table 15, which indicates that the implementation of AAQS significantly improves public health in terms of both physical health and mental health, and our results are consistent. Table 16 shows that the mediation effect is still significant, which is in line with previous conclusions. As a result, our findings are consistent after we control income and consumption.

#### 4.4.5. Control of Family Fixed Effect

We here employ family tracked survey data, and to further check the robustness of our model, in addition to city and year fixed effects, we control the family fixed effect in this section. Empirical results are presented in Table 17 and Table 18. Table 17 shows the results for baseline regressions, and the results of mediation effect are reported in Table 18.

The coefficients of EID are significantly positive in columns 1 to 4 in Table 17, which indicates that the EID significantly improves public health in terms of both physical health and mental health, and our results are consistent. Table 18 shows that the mediation effect is still significant, which is in line with previous conclusions. As a result, our findings are consistent after we control the family fixed effect.

## 5. Conclusions

This paper assesses the impact of environmental information disclosure on public health based on the implementation of the new Ambient Air Quality Standards in China. Using data from the China Health and Retirement Longitudinal Survey (CHARLS) for 2011, 2013, and 2015, this paper reaches the following conclusions.

Environmental information disclosure largely improves both physical health and mental health. Moreover, we further investigate the air pollution channel; the empirical results show that EID can reduce the concentration of PM_2.5_, and public health increases as the concentration of PM_2.5_ decreases. In addition, in terms of individual characteristics, the impact of EID is larger for men, people living in the countryside and those older than 60. In terms of the heterogeneity of cities, the impact of EID is larger in cities with higher public environmental concerns and more pronounced in core cities. For regional heterogeneity, the impact of the EID on physical health is more pronounced in more developed regions, whereas the impact of the EID on mental health is higher in less developed regions.

Our findings reveal that the implementation of the AAQS has a great impact on public health, which indicates that governments should implement more monitoring stations, especially in the countryside areas of China. Due to the problem of their energy consumption structure, significant environmental issues are present in these areas, and most people who live there long-term are usually older people. The implementation of the AAQS would be more beneficial for them.

## Figures and Tables

**Table 1:** The names of cities selected by the AAQS in 2012, 2013, and 2014.

Year	Name of Cities
2012	Beijing, Tianjin, Shijiazhuang, Tangshan, Qinhuangdao, Handan, Xingtai, Baoding, Zhangjiakou, Chengde, Cangzhou, Langfang, Hengshui, Taiyuan, Huhehaote, Shenyang, Dalian, Changchun, Harbin, Shanghai, Nanjing, Wuxi, Xuzhou, Changzhou, Suzhou, Nantong, Lianyungang, Huaian, Yancheng, Yangzhou, Zhenjiang, Taizhou, Suqian, Hangzhou, Ningbo, Wenzhou, Jiaxing, Huzhou, Shaoxing, Jinhua, Quzhou, Zhoushan, Taizhou, Lishui, Hefei, Fuzhou, Xiamen, Nanchang, Jinan, Qingdao, Zhengzhou, Wuhan, Changsha, Guangzhou, Shenzhen, Zhuhai, Foshan, Jiangmen, Zhaoqing, Huizhou, Dongguan, Zhongshan, Nanning, Haikou, Chongqing, Chengdu, Guiyang, Kunming, Lhasa, Xian, Lanzhou, Xining, Yinchuan, Urumqi
2013	Datong, Yangquan, Changzhi, Linfen, Baotou, Chifeng, Erdos, Anshan, Fushun, Benxi, Dandong, Jinzhou, Yingkou, Panjin, Huludao, Qiqihar, Daqing, Mudanjiang, Wuhu, Maanshan, Quanzhou, Jiujiang, Zibo, Zaozhuang, Dongying, Yantai, Weifang, Jining, Taian, Weihai, Rizhao, Linyi, Dezhou, Liaocheng, Binzhou, Heze, Kaifeng, Luoyang, Pingdingshan, Anyang, Jiaozuo, Sanmenxia, Yichang, Jingzhou, Zhuzhou, Xiangtan, Yueyang, Changde, Zhangjiajie, Shaoguan, Shantou, Zhanjiang, Maoming, Meizhou, Shanwei, Heyuan, Yangjiang, Qingyuan, Chaozhou, Jieyang, Yunfu, Liuzhou, Guilin, Beihai, Sanya, Zigong, Panzhihua, Luzhou, Deyang, Mianyang, Nanchong, Yibin, Zunyi, Qujing, Yuxi, Tongchuan, Baoji, Xianyang, Weinan, Yanan, Jiayuguan, Jinchang, Shizuishan, Karamay, Laiwu
2014	Jincheng, Shuozhou, Jinzhong, Yuncheng, Xinzhou, Lvliang, Wuhai, Tongliao, Hulunbeier, Bayan Nur, Ulaanchab, Fuxin, Liaoyang, Tieling, Chaoyang, Jilin, Siping, Liaoyuan, Tonghua, Baishan, Songyuan, Baicheng, Jixi, Hegang, Shuangyashan, Yichun, Jiamusi, Qitaihe, Hehe, Suihua, Bengbu, Huainan, Huabei, Tongling, Anqing, Huangshan, Chuzhou, Fuyang, SuZhou, Liuan, Bozhou, Chizhou, Xuancheng, Putian, Sanming, Zhangzhou, Nanping, Longyan, Ningde, Jingdezhen, Pingxiang, Xinyu, Yingtan, Ganzhou, jian, Yichun, Fuzhou, Shangrao, Hebi, xinxiang, Puyang, Xuchang, Luohe, Nanyang, Shangqiu, Xinyang, Zhoukou, Zhumadian, Huangshi, Shiyan, Xiangyang, Ezhou, Jingmen, Xiaogan, Huanggang, Xianning, Suizhou, Xiantao, Qianjiang, Tianmen, Shennongjia Forestry Area, Hengyang, Shaoyang, Yiyang, Chenzhou, Yongzhou, Huaihua, Loudi, Wuzhou, Fangchenggang, Qinzhou, Guigang, Yulin, Baise, Hezhou, Hechi, Laibin, Chongzuo, Sansha, Danzhou, Wuzhishan, Qionghai, Wenchang, Wanning, Dongfang, Guangyuan, Suining, Neijiang, Leshan, Meishan, Guangan, Dazhou, Yaan, Bazhong, Ziyang, Liupanshui, Anshun, Bijie, Tongren, Baoshan, Zhaotong, Lijiang, Puer, Lincang, Shigatse, Changdu, Linzhi, Shannan, Nagqu, Hanzhong, Yulin, Ankang, Shangluo, Baiyin, Tianshui, Wuwei, Zhangye, Pingliang, Jiuquan, Qingyang, Dingxi, Longnan, Haidong, Wuzhong, Guyuan, Zhongwei, Turpan, Hami, Shihezi, Aral, Tumushuk, Wujiaqu, Beitun, Tiemenguan, Shuanghe, Kekodara, Kunyu, Huyang River, Chaohu

**Table 2:** Measurement of public health.

Public Health	Indicators	Range of Values	Properties
Physical health	Acute shock	[0,3]	Negative
	Chronic shock	[0,9]	Negative
Mental health	Situational memory	[0,20]	Positive
	Mental cognition	[0,12]	Positive
	Depression self-assessment	[10,40]	Negative

**Table 3:** Descriptive statistics.

Variables		Variable Description	N	Mean	Min	Max	Std
Dependent variable	Physical health	Physical health level	69,758	0.970	0.222	1	0.073
	Mental health	Mental health level	69,758	0.471	0.059	1	0.149
Independent variable	EID	Implementation of EID	69,758	0.612	0	1	0.487
	PM_2.5_	Logarithmic value of PM_2.5_ concentration	69,758	0.471	0.059	1	0.149
	PEC	Logarithmic values of the Baidu index	69,758	3.749	1.692	4.641	0.424
Control variable	lnage	Logarithmic value of age	69,758	4.092	3.807	4.771	0.162
	gender	Male = 1, female = 0	69,758	0.516	0	1	0.500
	marital	Married = 1, otherwise = 0	69,758	0.863	0	1	0.344
	residence	Rural = 1, urban = 0	69,758	0.126	0	1	0.332
	lncost	Logarithmic value of hospitalization Costs	69,758	1.045	0	14.152	2.811
	toilet	No toilet = 0, otherwise = 1	69,758	0.778	0	1	0.416
	water	No running water = 0, otherwise = 1	69,758	0.731	0	1	0.444

**Table 4:** Impact of environmental information disclosure on public health.

Variables	Physical Health		Mental Health	
	(1)	(2)	(3)	(4)
EID	0.039 ***	0.041 ***	0.015 ***	0.009 ***
	(0.001)	(0.001)	(0.001)	(0.001)
lnage		−0.023 ***		0.195 ***
		(0.002)		(0.004)
gender		−0.003 ***		0.053 ***
		(0.001)		(0.001)
marital		−0.001		−0.036 ***
		(0.001)		(0.002)
residence		−0.018 ***		−0.036 ***
		(0.001)		(0.002)
lncost		−0.002 ***		0.002 ***
		(0.000)		(0.000)
toilet		0.001		0.016 ***
		(0.001)		(0.001)
water		0.003 ***		0.020 ***
		(0.001)		(0.001)
C	0.946 ***	1.046 ***	0.462 ***	−0.407 ***
	(0.001)	(0.008)	(0.001)	(0.018)
City FE	Y	Y	Y	Y
Year FE	Y	Y	Y	Y
Observation	69,758	69,758	69,758	69,758
F	3858.434	563.852	177.037	820.614
R2	0.081	0.096	0.069	0.173

Note: (1) *** denotes significant at the 1% level. (2) High-dimensional fixed-effects methods are used to control city FE effects and year FE effects. (3) Individual-level cluster robust standard errors are reported in parentheses.

**Table 5:** Impact of environmental information disclosure on different health indicators.

Variables	Acute Shock	Chronic Shock	Situational Memory	Mental Cognition	Depression Self-Assessment
	(1)	(2)	(3)	(4)	(5)
EID	−0.050 ***	−0.597 ***	0.145 ***	0.151	−0.253 ***
	(0.002)	(0.007)	(0.031)	(0.132)	(0.041)
C	−0.168 ***	1.360 ***	24.682 ***	14.485 ***	23.791 ***
	(0.025)	(0.076)	(0.422)	(0.759)	(0.541)
Control	Y	Y	Y	Y	Y
City FE	Y	Y	Y	Y	Y
Year FE	Y	Y	Y	Y	Y
Observation	75,816	75,816	75,816	75,816	75,816
F	131.068	982.150	632.131	52.093	205.546
R2	0.036	0.129	0.129	0.672	0.064

Note: (1) *** denotes significant at the 1% level. (2) High-dimensional fixed-effects methods are used to control city FE effects and year FE effects. (3) Individual-level cluster robust standard errors are reported in parentheses.

**Table 6:** Mechanism of environmental information disclosure affecting public health.

Variables	PM_2.5_	Physical Health	Mental Health
	(1)	(2)	(3)
EID	−0.201 ***	0.006 ***	0.004 ***
	(0.013)	(0.000)	(0.001)
PM_2.5_		−0.045 ***	−0.080 ***
		(0.002)	(0.005)
C	3.872 ***	1.747 ***	−0.765 ***
	(0.008)	(0.040)	(0.124)
Control	Y	Y	Y
City FE	Y	Y	Y
Year FE	Y	Y	Y
Sobel Z-score		−10.88	22.89
Observation	69,758	69,758	69,758
F	254.016	275.123	449.077
R2	0.907	0.456	0.752

Note: (1) *** denotes significant at the 1% level. (2) High-dimensional fixed-effects methods are used to control city FE effects and year FE effects. (3) Individual-level cluster robust standard errors are reported in parentheses.

**Table 7:** Differences in the effect of EID on public health by gender.

Variables	Physical Health		Mental Health	
	Male	Female	Male	Female
	(1)	(2)	(3)	(4)
EID	0.044 ***	0.038 ***	0.013 ***	0.004 ***
	(0.001)	(0.001)	(0.001)	(0.001)
C	1.039 ***	1.041 ***	−0.512 ***	−0.145 ***
	(0.011)	(0.010)	(0.025)	(0.025)
SUR test	23.24 ***		8.42 ***	
Control	Y	Y	Y	Y
City FE	Y	Y	Y	Y
Year FE	Y	Y	Y	Y
Observation	35,976	33,782	35,976	33,782
F	350.34	296.09	542.54	188.53
R2	0.103	0.092	0.214	0.095

Note: (1) *** denotes significant at the 1% level. (2) High-dimensional fixed-effects methods are used to control city FE effects and year FE effects. (3) Individual-level cluster robust standard errors are reported in parentheses.

**Table 8:** Differences in the effect of EID on the public health by residence.

Variables	Physical Health		Mental Health	
	Rural	Urban	Rural	Urban
	(1)	(2)	(3)	(4)
EID	0.079 ***	0.038 ***	0.006	0.009 ***
	(0.003)	(0.001)	(0.004)	(0.001)
C	1.047 ***	1.044 ***	−0.187 ***	−0.448 ***
	(0.025)	(0.008)	(0.047)	(0.019)
SUR test	203.99 ***		6.57 **	
Control	Y	Y	Y	Y
City FE	Y	Y	Y	Y
Year FE	Y	Y	Y	Y
Observation	8,782	60,976	8,782	60,976
F	114.70	533.54	59.47	924.37
R2	0.173	0.087	0.119	0.177

Note: (1) *** and ** denote significant at the 1% level and 5% level. (2) High-dimensional fixed-effects methods are used to control city FE effects and year FE effects. (3) Individual-level cluster robust standard errors are reported in parentheses.

**Table 9:** Differences in the effect of EID on public health by age.

Variables	Physical Health		Mental Health	
	45–60	Above 60	45–60	Above 60
	(1)	(2)	(3)	(4)
EID	0.037 ***	0.047 ***	0.006 ***	0.015 ***
	(0.001)	(0.001)	(0.001)	(0.002)
C	0.953 ***	0.945 ***	0.402 ***	0.405 ***
	(0.002)	(0.002)	(0.005)	(0.004)
SUR test	60.95 ***		10.48 **	
Control	Y	Y	Y	Y
City FE	Y	Y	Y	Y
Year FE	Y	Y	Y	Y
Observation	36,838	32,920	36,838	32,920
F	350.91	299.68	130.56	405.65
R2	0.097	0.098	0.094	0.176

Note: (1) *** and ** denote significant at the 1% level and 5% level. (2) High-dimensional fixed-effects methods are used to control city FE effects and year FE effects. (3) Individual-level cluster robust standard errors are reported in parentheses.

**Table 10:** Moderating effects of public environmental concerns.

Variables	Physical Health		Mental Health	
	(1)	(2)	(3)	(4)
PEC	0.047 ***	−0.021 ***	0.046 ***	0.036 ***
	(0.008)	(0.002)	(0.009)	(0.004)
EID		0.060 ***		0.030
		(0.008)		(0.022)
EID×PEC		0.007 ***		0.009 ***
		(0.001)		(0.002)
C	0.538 ***	2.111 ***	0.039	−2.071 ***
	(0.074)	(0.037)	(0.079)	(0.088)
Control	Y	Y	Y	Y
City FE	Y	Y	Y	Y
Year FE	Y	Y	Y	Y
Observation	69,758	69,758	69,758	69,758
F	33.659	223.202	29.715	325.673
R2	0.031	0.451	0.063	0.750

Note: (1) *** denotes significant at the 1% level. (2) High-dimensional fixed-effects methods are used to control city FE effects and year FE effects. (3) Individual-level cluster robust standard errors are reported in parentheses.

**Table 11:** Differences in the effect of environmental information disclosure on public health by urban administrative rank.

Variables	Physical Health		Mental Health	
	Non-Ordinary Prefecture-Level Cities	Ordinary Prefecture-Level Cities	Non-Ordinary Prefecture-Level Cities	Ordinary Prefecture-Level Cities
	(1)	(2)	(4)	(5)
EID	0.075 ***	0.037 ***	0.018 ***	0.011 ***
	(0.002)	(0.001)	(0.003)	(0.001)
C	0.951 ***	0.967 ***	−0.074 ***	−0.170 ***
	(0.011)	(0.013)	(0.024)	(0.026)
Control	Y	Y	Y	Y
City FE	Y	Y	Y	Y
Year FE	Y	Y	Y	Y
Observation	11,448	58,310	11,448	58,310
F	173.638	413.536	84.425	768.846
R2	0.214	0.081	0.119	0.183

Note: (1) *** denotes significant at the 1% level. (2) High-dimensional fixed-effects methods are used to control city FE effects and year FE effects. (3) Individual-level cluster robust standard errors are reported in parentheses.

**Table 12:** Differences in the effect of environmental information disclosure on public health by economic development level.

Variables	Physical Health			Mental Health		
	Eastern	Central	Western	Eastern	Central	Western
	(1)	(2)	(3)	(4)	(5)	(6)
EID	0.047 ***	0.039 ***	0.039 ***	0.003 ***	0.008 ***	0.015 ***
	(0.001)	(0.001)	(0.001)	(0.000)	(0.002)	(0.002)
C	1.068 ***	1.052 ***	1.017 ***	−0.424 ***	−0.454 ***	−0.348 ***
	(0.012)	(0.014)	(0.014)	(0.030)	(0.031)	(0.031)
Control	Y	Y	Y	Y	Y	Y
City FE	Y	Y	Y	Y	Y	Y
Year FE	Y	Y	Y	Y	Y	Y
Observation	24,020	22,953	22,785	24,020	22,953	22,785
F	219.190	168.099	187.401	263.913	282.139	283.258
R2	0.122	0.079	0.093	0.143	0.160	0.191

Note: (1) *** denotes significant at the 1% level. (2) High-dimensional fixed-effects methods are used to control city FE effects and year FE effects. (3) Individual-level cluster robust standard errors are reported in parentheses.

**Table 13:** Re-estimation based on PSM-DID.

Variables	Physical Health	Mental Health
	(1)	(2)
EID	0.057 ***	0.009 ***
	(0.003)	(0.003)
lnage	−0.014 ***	0.205 ***
	(0.002)	(0.005)
gender	−0.000	0.054 ***
	(0.001)	(0.002)
marital	−0.000	−0.036 ***
	(0.001)	(0.002)
residence	−0.012 ***	−0.037 ***
	(0.001)	(0.002)
lncost	−0.002 ***	0.002 ***
	(0.000)	(0.000)
toilet	0.000	0.014 ***
	(0.001)	(0.002)
water	−0.002 ***	0.025 ***
	(0.001)	(0.002)
C	0.992 ***	−0.453 ***
	(0.008)	(0.022)
City FE	Y	Y
Year FE	Y	Y
Observation	44,929	44,929
F	129.765	585.025
R2	0.083	0.189

Note: (1) *** denotes significant at the 1% level. (2) High-dimensional fixed-effects methods are used to control city FE effects and year FE effects. (3) Individual-level cluster robust standard errors are reported in parentheses.

**Table 14:** Re-estimation using different dependent variables.

Variables	Health	Health
	(1)	(2)
EID	0.464 ***	0.448 ***
	(0.009)	(0.009)
lnage		−0.313 ***
		(0.035)
gender		−0.173 ***
		(0.010)
marital		−0.056 ***
		(0.017)
residence		0.265 ***
		(0.016)
lncost		−0.053 ***
		(0.002)
toilet		0.059 ***
		(0.012)
water		0.107 ***
		(0.012)
C	1.512 ***	3.011 ***
	(0.006)	(0.152)
City FE	Y	Y
Year FE	Y	Y
Observation	69,758	69,758
F	2676.329	595.881
R2	0.066	0.094

Note: (1) *** denotes significant at the 1%. (2) High-dimensional fixed-effects methods are used to control city FE effects and year FE effects. (3) Individual-level cluster robust standard errors are reported in parentheses.

**Table 15:** The results of baseline regressions after adding new control variables.

	Physical_Health	Physical_Health	Mental_Health	Mental_Health
	(1)	(2)	(3)	(4)
EID	0.039 ***	0.024 ***	0.015 ***	0.014 ***
	(0.001)	(0.001)	(0.001)	(0.001)
lnage		−0.024 ***		0.194 ***
		(0.002)		(0.004)
gender		−0.003 ***		0.053 ***
		(0.001)		(0.001)
marital		−0.001		−0.036 ***
		(0.001)		(0.002)
residence		−0.016 ***		−0.038 ***
		(0.001)		(0.002)
lncost		−0.002 ***		0.002 ***
		(0.000)		(0.000)
toilet		−0.000		0.016 ***
		(0.001)		(0.001)
water		0.003 ***		0.020 ***
		(0.001)		(0.001)
income		−0.036 ***		0.050 ***
		(0.002)		(0.003)
consumption		0.117 ***		−0.075 ***
		(0.003)		(0.004)
C	0.946 ***	0.256 ***	0.462 ***	−0.133 ***
	(0.001)	(0.026)	(0.001)	(0.045)
City FE	Y	Y	Y	Y
Year FE	Y	Y	Y	Y
Observation	69,758	69,758	69,758	69,758
F	3858.434	616.222	177.037	715.535
R^2^	0.081	0.125	0.069	0.177

Note: (1) *** denotes significant at the 1% level. (2) High-dimensional fixed-effects methods are used to control city FE effects and year FE effects. (3) Individual-level cluster robust standard errors are reported in parentheses.

**Table 16:** The results of mediation effect after adding new control variables.

	Lnpm25	Physical_Health	Mental_Health
	(1)	(2)	(3)
EID	−0.201 ***	0.001 *	0.006 ***
	(0.013)	(0.001)	(0.002)
PM_2.5_		−0.040 ***	−0.075 ***
		(0.002)	(0.005)
lnage		−0.133***	0.325 ***
		(0.010)	(0.027)
gender		−0.002	0.028 **
		(0.003)	(0.014)
marital		0.007 ***	−0.027 ***
		(0.002)	(0.004)
residence		−0.001	−0.008 ***
		(0.001)	(0.002)
lncost		−0.001 ***	0.001 ***
		(0.000)	(0.000)
toilet		0.000	0.027 ***
		(0.001)	(0.002)
water		−0.002 **	0.038 ***
		(0.001)	(0.002)
income		−0.017 ***	0.023 ***
		(0.002)	(0.004)
consumption		0.019 ***	−0.049 ***
		(0.003)	(0.005)
C	3.872 ***	1.601 ***	−0.379 ***
	(0.008)	(0.045)	(0.132)
City FE	Y	Y	Y
Year FE	Y	Y	Y
Sobel Z-score		−12.58	24.96
Observation	68,719	48,359	48,359
F	254.016	228.666	379.502
R2	0.907	0.458	0.752
F	254.016	228.666	379.502

Note: (1) ***, **, and * denote significant at the 1% level, 5% level, and 10% level. (2) High-dimensional fixed-effects methods are used to control city FE effects and year FE effects. (3) Individual-level cluster robust standard errors are reported in parentheses.

**Table 17:** The results of baseline regressions after controlling family fixed effect.

	Physical_Health	Physical_Health	Mental_Health	Mental_Health
	(1)	(2)	(3)	(4)
EID	0.016 ***	0.005 ***	0.041 ***	0.019 ***
	(0.000)	(0.001)	(0.001)	(0.002)
lnage		−0.104 ***		0.238 ***
		(0.006)		(0.015)
gender		−0.006 ***		0.055 ***
		(0.000)		(0.001)
marital		0.008 ***		−0.014 ***
		(0.002)		(0.004)
residence		−0.003 ***		−0.002
		(0.001)		(0.002)
lncost		−0.002 ***		0.002 ***
		(0.000)		(0.000)
toilet		0.001		0.027 ***
		(0.001)		(0.002)
water		−0.003 ***		0.041 ***
		(0.001)		(0.002)
income		−0.041 ***		0.068 ***
		(0.002)		(0.004)
consumption		0.023 ***		−0.057 ***
		(0.003)		(0.006)
C	0.968 ***	1.549 ***	0.442 ***	−0.704 ***
	(0.000)	(0.036)	(0.001)	(0.080)
City FE	Y	Y	Y	Y
Year FE	Y	Y	Y	Y
Family FE	Y	Y	Y	Y
Observation	69,758	69,758	69,758	69,758
F	1107.604	183.818	798.714	443.252
R^2^	0.404	0.427	0.355	0.409

Note: (1) *** denotes significant at the 1% level. (2) High-dimensional fixed-effects methods are used to control city FE effects and year FE effects. (3) Individual-level cluster robust standard errors are reported in parentheses.

**Table 18:** The results of mediation effect after controlling family fixed effect.

	lnpm25	Physical_Health	Mental_Health
	(1)	(2)	(3)
EID	−0.201 ***	0.001 *	0.010 ***
	(0.013)	(0.000)	(0.002)
PM_2.5_		−0.050 ***	−0.100 ***
		(0.002)	(0.005)
lnage		−0.054 ***	0.136 ***
		(0.006)	(0.015)
gender		−0.004 ***	0.051 ***
		(0.000)	(0.001)
marital		0.005 **	−0.008 *
		(0.002)	(0.004)
residence		−0.002	−0.005 **
		(0.001)	(0.002)
lncost		−0.002 ***	0.002 ***
		(0.000)	(0.000)
toilet		0.001	0.026 ***
		(0.001)	(0.002)
water		−0.002 **	0.038 ***
		(0.001)	(0.002)
income		−0.021 ***	0.030 ***
		(0.002)	(0.004)
consumption		0.020 ***	−0.052 ***
		(0.003)	(0.006)
C	3.872 ***	1.314 ***	0.398 ***
	(0.008)	(0.037)	(0.098)
City FE	Y	Y	Y
Year FE	Y	Y	Y
Family FE	Y	Y	Y
Sobel Z-score		-15.23	20.38
Observation	68,719	48,359	48,359
F	254.016	228.666	379.502
R2	0.907	0.434	0.413
F	254.016	181.359	434.040

Note: (1) ***, **, and * denote significant at the 1% level, 5% level, and 10% level. (2) High-dimensional fixed-effects methods are used to control city FE effects and year FE effects. (3) Individual-level cluster robust standard errors are reported in parentheses.

## Data Availability

Data will be made available on request.
